# Predictors of engaging in voluntary work during the COVID-19 pandemic: analyses of data from 31,890 adults in the UK

**DOI:** 10.1177/1757913921994146

**Published:** 2021-04-15

**Authors:** HW Mak, D Fancourt

**Affiliations:** Department of Behavioural Science and Health, Institute of Epidemiology and Health Care, University College London, London, UK; Department of Behavioural Science and Health, Institute of Epidemiology and Health Care, University College London, 1-19 Torrington Place, London WC1E 7HB, UK

**Keywords:** volunteer, COVID-19, demography, socioeconomic factors, personality, psychosocial factors

## Abstract

**Aims:**

As the COVID-19 pandemic has grown internationally, there has been an increased need for volunteers. This study aimed to identify the predictors of volunteering including demographic backgrounds, socio-economic characteristics, personality, and psychosocial factors.

**Methods:**

Data were analysed from 31,890 adults in the UK COVID-19 Social Study run by the University College London – a longitudinal study focusing on the psychological and social experiences of adults living in the UK during the COVID-19 pandemic. Tetrachoric factor analysis was applied to identify latent categories of voluntary work. Multivariate logistic regression was used to identity predictors for volunteering and change in volunteering behaviours since before the COVID-19 pandemic.

**Results:**

Three types of volunteering during the pandemic were identified as follows: formal volunteering, social action volunteering, and neighbourhood volunteering. Regression analysis showed that the pattern of voluntary work was structured by demographic backgrounds, socio-economic factors, personality, and psychosocial factors.

**Conclusion:**

The predictors of volunteering during the pandemic may be slightly different from other non-emergency period.

## Introduction

As the Coronavirus (COVID-19) situation develops in the UK, there has been an increased need from volunteers to support individuals affected by the virus or to assist in the delivery of essential activities. Voluntary work has included shopping, packing and delivering food, medicine and supplies, as well as driving healthcare staff around, helping with food banks and homeless services, fund-raising and making donations, and providing emotional support through telephone helplines to tackle loneliness and social isolation. This work has been coordinated by various bodies including third sector groups and community organisations who have mobilised to support efforts relating to the pandemic, as well as through the Royal Voluntary Service’s GoodSAM programme,^1^ which has had over 1 million people registering, and through self-organised COVID-19 mutual aid groups, which number over 3000 across the UK.^2^

Previous research into motivations for community volunteering (e.g. fund-raising, supporting local charities, and assisting in nurseries or care homes) has shown that motivations to volunteer include altruism,^3–5^ having a strong sense of purpose,^6–8^ a desire to enhance human capital (e.g. through gaining employment experience or developing skills),^3,5^ improvement of mental and physical health,^6,9^ and wanting to feel empowered and in control.^6^ Furthermore, social rewards (e.g. engagement in group activities)^3,10^ and social recognition and approval of volunteering from others (particularly when it is perceived to be essential to the welfare and wellbeing of others)^11^ may also be important factors for encouraging volunteering. But an important question is whether volunteering is also predicted by demographic or socio-economic factors or other traits.

Research on characteristics of volunteers has mainly focused on community volunteers taking part during non-emergency situations. These studies have demonstrated that females, married people, people with children, people with higher educational levels and income, and people living in rural areas are more likely to take part in voluntary work.^3,12–15^ People over the age of 50 years have been found in some studies to be more likely to volunteer than young people,^3^ although other studies have also suggested that participation in voluntary work can decline with age.^12–14^ Previous studies have also shown that people with fewer depressive symptoms have a higher tendency to engage in volunteering;^13^ however, the association may vary by age.^16^ In addition, a large personal social network, religious participation, and personality (in particular, traits of agreeableness and extraversion) have also been identified as predictors of volunteering behaviours.^12,13,15,17^ In relation to volunteering following an international disaster, the literature suggests that the characteristics of the volunteers are similar to those who take part during non-emergency situations. For instance, a study focusing on international disaster volunteers following the 2010 Haiti earthquake found that the volunteer population was mainly made up of younger adults, women, people who were highly educated, and those with previous volunteering experience.^18^ While it is reasonable to assume that the characteristics of volunteers are relatively similar across time, volunteering during a pandemic may attract different demographic groups. For instance, a study on the willingness to volunteer among university staff and students during an influenza pandemic found that while older adults and people with previous voluntary activities were more willing to volunteer (in line with other studies), there were other pandemic-related factors influencing the decision to volunteer (such as risk perception and general knowledge regarding pandemic influenza).^19^ As a result, in the context of the COVID-19 pandemic, a desire to provide support during a national crisis may have helped to engage people who would not usually volunteer, while individuals not working (e.g. those on furlough) may have had more time available to volunteer. However, people with children who are unable to go to school may have been unable to provide as much time, and older adults who may have been at higher risk from the virus may have been unable to engage in certain volunteering activities.

Understanding who is likely to engage in volunteering during the COVID-19 pandemic could support future efforts to recruit more volunteers in the coming months, as well as help sustain local health systems and in planning for future epidemics. Therefore, this study was designed to (1) identify the latent categories of volunteering people engaged in during the COVID-19 pandemic from a longlist of volunteering activities and (2) identify how volunteering behaviours varied depending on a rich panel of demographic backgrounds, socio-economic characteristics, personality, and psychosocial factors.

## Methods

### Participants

Data were drawn from the UK COVID-19 Social Study run by the University College London – a longitudinal study focusing on the psychological and social experiences of over 70,000 adults (aged 18+ years) living in the UK during the COVID-19 pandemic. The study commenced on 21 March 2020 and involves online weekly data collection from participants for the duration of the COVID-19 pandemic in the UK. The study is not random and therefore is not representative of the UK population. But it does contain a heterogeneous sample that was recruited using three primary approaches. First, snowballing was used, including promoting the study through existing networks and mailing lists (including large databases of adults who had previously consented to be involved in health research across the UK), print and digital media coverage, and social media. Second, more targeted recruitment was undertaken focusing on (1) individuals from a low-income background, (2) individuals with no or few educational qualifications, and (3) individuals who were unemployed. Third, the study was promoted via partnerships with third sector organisations to vulnerable groups, including adults with pre-existing mental health conditions, older adults, carers, and people experiencing domestic violence or abuse. The study was approved by the UCL Research Ethics Committee (12467/005), and all participants gave informed consent. A full protocol for the study is available online at: www.COVIDSocialStudy.org.

Volunteering behaviours were asked for as a one-off module in week 7 of data collection from 21 April 2020 to 3 May 2020 (when the UK was in a national lockdown), with 35,471 participants completing a survey within these dates and thus providing data. As the survey involved mandatory question responses, there were no missing data on the volunteering questions. Within the sample, 3581 participants opted not to provide details on gender, ethnicity, or household income, leaving a final sample size with complete data of 31,890 participants.

### Measures

We considered a set of 13 variables on volunteering behaviours in the past month (see Table 1 for a full list). Responses were measured on a 5-point scale, ranging from ‘none’ to ‘everyday’, which were collapsed into a binary indicator of engaged versus did not engage. We also asked participants to rate whether their volunteering levels were less than usual (prior to the COVID-19 pandemic), about the same as usual, or more than usual. In June/July, this question was repeated, asking respondents to compare their frequency of volunteering in June/July (when the coronavirus restrictions were more relaxed) with the frequency in April/May to assess whether any changes in volunteering behaviours during lockdown had been maintained.

To understand how types of volunteering varied across personal characteristics and backgrounds, we considered a rich set of demographic factors, socio-economic factors, personality traits, and psychosocial factors including respondents’ age, gender, ethnicity, partnership status, living arrangement, number of children in the household, and living area. Socio-economic factors included employment status, educational level, household income, housing space, and whether respondents were keyworkers. Our model also considered the Big 5 personalities which are comprised of extraversion, neuroticism, openness, conscientiousness, and agreeableness. Finally, we included two psychosocial measures: social support (a modified version of the short form of Perceived Social Support Questionnaire (F-SozU K-6)) and size of social network. We also asked participants if they had any diagnosed mental health conditions, or any diagnosed physical condition or disability. The coding of each variable is shown in Supplementary Material (‘Methods – coding of the covariates’).

### Analyses

To identify the underlying latent categories of voluntary work, we ran a factor analysis of the matrix of tetrachoric correlations using all the volunteering measures (Kaiser–Meyer–Olkin (KMO) = 83.9). Kaiser’s criterion of eigenvalues >1, inspection of a scree plot, and oblique and orthogonal rotations indicated a three-factor structure. ‘Formal volunteering’ included volunteering with existing organisations or within formal volunteering structures, which usually required a higher degree of commitment (e.g. volunteering with deliveries or providing lifts to National Health Service (NHS) staff). ‘Social action volunteering’ included providing donations or more specialised pro bono support. This type of volunteering often involved the Internet and hence is not restricted to local activities. ‘Neighbourhood support’ included supporting others locally (e.g. running errands and making meals for others; Table 1). We generated a binary indicator of whether respondents had ever engaged in any activity within each of the three categories.

We then used multivariate logistic regression to calculate the odds ratio (OR) and 95% confidence intervals (CIs) that participants engaged in each type of volunteering behaviour based on predictor variables, and used multinomial logistic regression to estimate the relative risk ratio (RRR) to understand if this level of volunteering was less or more than usual. Four sets of models were run for each type of volunteering. Model 1 examined the association between demographic factors and volunteering. In Model 2, we additionally added socio-economic factors to the model. Model 3 involved Model 2 + personality measures, while Model 4 involved Model 2 + psychosocial factors. We did not mutually adjust for personality and psychosocial factors due to collinearity. As a sensitivity analysis, we repeated all analyses excluding those who identified themselves as keyworkers.

To balance the data against population demographics, we weighted data to the proportion of gender, age, ethnicity, education, and country of living obtained from the Office for National Statistics.^20^ All analyses were carried out in Stata v16.1.

## Results

In our sample, the average age was 52 years (standard error (SE) = 15 years). 51% were females and 91% were of White ethnic. On average, 62% of the sample were in a relationship/married and cohabiting, 43% were in full-time employment or self-employed, 37% had a degree or above, and 21% identified themselves as keyworkers (Table 2).

### Demographic backgrounds

Older people were more likely than younger adults to participate in neighbourhood volunteering, with 1-year increase in age associated with 13% higher odds (Table 3). There was no difference in formal or social action volunteering by age. Females were more likely to engage in social action volunteering (31% higher odds) and neighbourhood volunteering (20% higher odds), but not formal volunteering. However, for ethnicity, the pattern was the opposite. People of White ethnicity had 35% lower odds of engaging in formal volunteering, but ethnicity did not predict other volunteering activities. Married and cohabitating couples were less likely to take part in neighbourhood volunteering than those who were not living with a spouse. People living alone were less likely to involve in social action and neighbourhood volunteering. Respondents living with children had 5% lower odds of participating in social action volunteering; however, they were more likely (16% higher odds) than those who were not living with children to take part in neighbourhood volunteering. Respondents who lived in urban areas were less likely to engage in volunteering activities, particularly in formal and neighbourhood volunteering. However, there was no difference in social action volunteering. Results were replicated when excluding keyworkers from analyses (Supplementary Table 2).

### Socio-Economic Factors

People who were currently employed or with other responsibilities (e.g. students) were more likely to engage in social action volunteering (Table 3). But no difference was found in other types of volunteering. Respondents with a household income higher than £30,000 per annum had 34% higher odds of engaging in social action volunteering, but income did not predict other types of volunteering. Education predicted all types of volunteering: respondents with a degree or higher qualification were two times the odds more likely to engage in formal and social action volunteering. Moreover, keyworkers had 45% and 34% higher odds of participating in formal and neighbourhood volunteering, respectively. No difference was found for social action volunteering by keyworker status. Housing space did not predict any volunteering activities. Results were replicated when excluding keyworkers from analyses (Supplementary Table 2).

### Personality

Individuals with higher scores in extraversion, openness, and agreeableness were more likely to engage in all types of activities (Table 3). Respondents who scored high in neuroticism were more likely to take part in social action volunteering but less likely in neighbourhood volunteering. However, those with higher scores in conscientiousness were more likely to engage in neighbourhood volunteering. No difference was found in formal volunteering by neuroticism or conscientiousness. Results were replicated when excluding keyworkers from analyses (Supplementary Table 2).

### Psychosocial Factors

Respondents with higher levels of social support and those with a larger social network were more likely to participate in all types of voluntary work (Table 3). Engagement in social action volunteering was associated with people with diagnosed mental health (16% higher odds) or physical illness (8% higher odds) condition. People with diagnosed mental health conditions had 23% higher odds of engaging in formal volunteering, while those with physical illness condition had 27% lower odds of engaging in neighbourhood volunteering. Results were replicated when excluding keyworkers from analyses (Supplementary Table 2).

### Amount Of Volunteering

When comparing the levels of people’s volunteering during and before the pandemic, 12% of respondents reported that they had increased their participation in volunteering during lockdown compared to prior to the pandemic, 65% had about the same amount of engagement levels before and during the pandemic, and 23% decreased their engagement.

When re-measuring in June/July, 7% of people who reported increasing their volunteering during lockdown had further increased their engagement 3 months later. Conversely, 6% of respondents reported that their volunteering decreased during lockdown had further lowered 3 months later (Table 4).

When comparing the amount of volunteering during the COVID-19 pandemic to the amount during usual times (i.e. prior to the pandemic), results show that older adults, people with higher educational qualifications, and those with more social support were doing more voluntary work during the pandemic than before, whereas those living in urban areas were less likely to have increased their volunteering (Supplementary Table 3). People of White ethnicity were less likely to have decreased their volunteering, as were people who were employed, while people who were divorced or living apart from their spouse, people who were neurotic, and people with a physical health condition were more likely to have decreased their volunteering. Finally, some other factors were associated with a change from usual patterns, but this change could involve either an increase or a decrease in volunteering (including being female, extraversion, openness, agreeableness, social network size, and having a diagnosed mental illness).

## Discussion

The COVID-19 pandemic led to an increase in volunteering behaviours across the UK. This study suggests that there were three main types of volunteering during the pandemic: formal volunteering, social action volunteering, and neighbourhood volunteering. Notably, only a few factors predicted all types of volunteering behaviours (high educational qualifications, extraversion, openness, agreeableness, social support, and size of social network). However, many factors predicted specific types of volunteering, including older age, being female, having an ethnic minority background, not living with a spouse, living with others, living in a rural location, being employed or having other responsibilities (e.g. students/homemakers), being a keyworker, and diagnosed health conditions. Overall, 12% of respondents reported that they had increased their participation in volunteering during lockdown and 26% of those maintained this higher volunteering or further increased it 3 months later even after lockdown had eased. Conversely, 23% of respondents reported decreasing their volunteering during lockdown, and of these, 17% reported that they had maintained these lower levels or had a further decrease in volunteering 3 months later. While older adults, people with higher educational qualifications, and those with more social support engaged more frequently during the pandemic, people who were divorced or living apart from their spouse, those who were neurotic, and people with a physical health condition engaged less.

A number of the predictive factors identified here align with well-known predictors of volunteering, such as being female, living with children, living in rural or remote area, having higher educational qualifications, and having higher household income.^3,12–15,21^ We also found that agreeableness and extraversion were associated with engagement in all types of voluntary work, whereas people with higher levels of neuroticism were less likely to volunteer, potentially due to concerns about catching the virus (as also shown in previous studies).^17,22^ Furthermore, in line with the literature, our results show that both social support and social network predicted all kinds of voluntary work.^23,24^ There are different possible explanations for this. Individuals with greater levels of social capital may be encouraged to volunteer through a stronger sense of social identity, social connectedness, and desire for social cooperation.^25^ These individuals may also be better connected with the needs of their communities and therefore more readily become aware of needs for volunteers. However, given that this is a cross-sectional study, the association between social support, social network, and volunteering might be bidirectional.

However, our results suggest that certain well-known predictors have not been as clear-cut as prior to the pandemic. For example, in contrast to other studies, our results also reveal that openness predicted all types of volunteering. It is plausible that people who are open to new ideas and experiences may be more inclined to volunteer in challenging projects during the pandemic as opposed to traditional voluntary work in usual time (prior to the outbreak of COVID-19). While health status has often been considered as one of the influential predictors of volunteering, results are less conclusive (possibly due to various definitions of health, e.g. self-rated health vs functional limitations vs chronic diseases).^12,16,22,26^ Nonetheless, our findings show that people with a diagnosed physical illness or disability had a lower odds of volunteering in neighbourhood support, and it is unsurprising given that many people with illnesses were considered more at risk of the virus. However, we also found that people with a diagnosed physical illness or disability were more likely to do social action volunteering, which could indicate a desire to support efforts, but in activities that can be done generally from one’s own home (e.g. participation in Internet research). Similarly, people with a diagnosed mental health condition were more likely to do either formal or social action volunteering than people without a diagnosed condition. A potential explanation for this is that volunteering may be used as a means of compensation for attenuated social relationships among those with a mental health condition.^16^

We also found some further results that are noteworthy given previous literature on these factors as predictors of volunteering has been mixed. For example, we found that older people were more likely to volunteer (in particular, engaging in neighbourhood volunteering), and more likely to have increased their volunteering specifically due to COVID-19, despite being designated as high risk. This is a clearer pattern than in some previous studies of volunteering that have found mixed results on the relationship between age and volunteering.^3,12^ This indicates that older adults might use volunteerism to fill a void created by physical and social distancing and to extend social relationships during lockdown when there were limited contacts with other family members or network members.^23^ Furthermore, while research into the association between ethnicity and participation was inconclusive,^12^ we found that people of White ethnic were less likely to participate in formal volunteering. No differences were found in other types of voluntary work; this may explain the heterogeneous results shown in other studies that did not differentiate between different types of volunteering activities. But people of White ethnic were less likely to have decreased their volunteering due to the COVID-19 pandemic, suggesting that their volunteering was a continuation of previous habits. While the association between employment status and volunteering is heterogeneous and inconsistent in the previous literature,^3,12^ we found that it was a strong predictor for social action voluntary work. However, there was no difference in other types of volunteering activities between those who were employed and who were unemployed, and people who were unemployed and seeking work did not show any differential patterns of change in their volunteering behaviours from people who were economically inactive (e.g. homemakers, students, and people who were retired or unable to work).

Therefore, overall this article showed some similarity with previous predictors of volunteering, but also some novel predictors during the COVID-19 pandemic. While we have outlined specific reasons why there might be variation in certain specific predictors above based on the context of the pandemic, there are also some broader explanations for why there were differences between volunteering patterns during COVID-19 and in previous circumstances relating to how the barriers to and enablers of volunteering changed. For example, if we consider people’s capabilities, opportunities, and motivations to volunteer using a behaviour change framework,^27^ the national drive for volunteers led by the NHS provided clear opportunities to engage and reduced the psychological capability barrier around whether people were aware of local opportunities to them; the proliferation of research that people could take part in from their own homes provided new physical opportunities along with low barriers relating to personal physical capability; and the social focus on mutual support provided new motivations to engage. While our analysis was based on a large, well-stratified sample weighted to population proportions and considered a rich set of predictors to estimate the types of voluntary work, the study is not without limitations. First, we looked at 13 specific types of volunteering and explored the factor structure of these items, but the list of volunteering types is not exhaustive, and other specific volunteering activities may have been omitted from the study. While we included ‘other volunteering’ as an item, it is possible that different definitions of volunteering could have led to different factor groupings, and therefore, we present the groupings here as indicators of latent classes of volunteering activities rather than definitive categories. Relatedly, some of the voluntary work may reflect a response to support the COVID-19 pandemic, whereas others may be activities that were considered to be more ‘general’ in which participants might have partaken prior to the pandemic (e.g. volunteering with childcare for a friend, relative, or neighbours). This study focused on these volunteering behaviours in the context of the pandemic, but this does not mean that such behaviours might not also have been in place before the pandemic and as such might reflect non-pandemic-related volunteering behaviours. Furthermore, due to data unavailability, we were unable to control for participants’ previous volunteering experience,^18,19^ religious beliefs, participation and affiliations,^12,13,15,28^ and friends’ or family’s involvement in voluntary work during the pandemic.^3^ All have been shown to help predict a person’s voluntary engagement. Although we asked participants to self-report diagnosed mental and physical health conditions, this study did not look at whether level of depression or anxiety symptoms predicted participation. Moreover, we asked about socio-demographic factors such as employment status and social network size at baseline, so these associations represent baseline associations. They do not indicate how participation in schemes such as furlough schemes might have affected volunteering, nor whether changes in frequency of social contact motivated greater volunteering. Future research is needed to investigate how changing circumstances during the pandemic differentially motivated individuals to volunteer, and to explore the impact of volunteering on trajectories of mental health during the COVID-19 pandemic.

Overall, this study suggests that many of the volunteers during the COVID-19 pandemic in the UK were people who fulfil the typical demographic profiles of volunteers in normal circumstances. This suggests that the results of this study have a relevance to non-emergency situations in highlighting the consistency of capabilities, opportunities, and motivations to volunteer among adults. However, other new groups were identified as likely to volunteer including people with mental and physical health conditions. Along with voluntary work playing a vital role in supporting individuals and communities, volunteering has numerous benefits for health and wellbeing (e.g. better self-rated health, reduced levels of depression, improved wellbeing, self-esteem, and quality of life).^29,30^ Therefore, exploring how these new groups of volunteers can be engaged and retained as volunteers beyond a pandemic is important for public health and to sustain local health systems as a whole. Future studies are also required specifically to understand the impact of volunteering during a national crisis as well as the factors predicting the duration of volunteering behaviours after the initial enthusiasm to provide support has declined. Nevertheless, these results give an insight into the profiles of individuals who could be targeted to engage further in volunteering should more demand arise during this or future pandemics.

## Supplementary Material

Supplementary Material

## Figures and Tables

**Table 1:** Tetrachoric factor analysis for types of volunteering during the COVID-19 pandemic in the UK

	Factor 1	Factor 2	Factor 3
	Formal volunteering	Social action volunteering	Neighbourhood support
Volunteering with childcare for a friend, relative, or neighbours			0.6280
Running errands for friends, relatives, or neighbours (e.g. collecting shopping and medication)			0.7686
Making meals for friends, relatives, or neighbours			0.7459
Volunteering with deliveries or providing lifts to NHS staff	0.6624		
Volunteering at a hospital, care home, or other healthcare facility	0.7668		
Volunteering taking part in research (other than this study)		0.3831	
Offering telephone support to others through a support line (e.g. Samaritans or GoodSAM)	0.6112		
Providing free accommodation to people affected by COVID-19 (e.g. NHS staff or people who are homeless)	0.5798		
Donating money to charities supporting COVID-19		0.6778	
Providing entertainment to others (e.g. via social media or YouTube) to boost morale		0.6994	
Providing pro bono support to businesses or projects		0.6031	
Other volunteering activity relating to COVID-19	0.6815		
Other volunteering activity NOT relating to COVID-19		0.5049	

NHS: National Health Service.

**Table 2:** Descriptive statistics of the sample (weighted; *N* = 31,890)

	% or mean (SE)
**Demographic backgrounds**	
Age (ranging from 18 to 106)	52.1 (15.3)
Female	51.4
Male	48.6
White ethnic	90.7
Ethnic minority	9.33
Single and never married	18.1
Divorced or widowed	13.8
In a relationship/married but living apart	6.48
In a relationship/married and cohabiting	61.6
Living alone	22.9
Not living alone	77.1
Number of children in the household (ranging from 0 to 10+)	0.38 (0.01)
Living in city/town	77.6
Living in village/hamlet/isolated dwelling	22.4
**Socio-economic position**	
Full-time employment/self-employed	43.3
Part-time employment	12.0
Student/retired/homemakers/unable to work due to disability	42.3
Unemployed and seeking work	2.42
Degree or above	37.4
A-levels	22.9
GCSE/post 16 vocational qualification	33.5
No qualification	6.32
Household income >£30,000	51.2
Household income <£30,000	48.8
Standard room/space households	97.1
Overcrowded households	2.89
Keyworkers	20.7
Not keyworkers	79.3
**Big five personalities (all standardised)**	
Extraversion	−0.04 (0.01)
Neuroticism	−0.06 (0.01)
Openness	−0.11 (0.01)
Conscientiousness	−0.06 (0.01)
Agreeableness	−0.04 (0.01)
**Psychosocial measures**	
Social support (ranging from 6 to 30)	21.5 (0.06)
Social network (defined as <3 friends vs 3+ friends)	69.5
Diagnosed mental health condition	18.2
Diagnosed physical health condition or disability	44.7

SE: standard error; GCSE: General Certificate of Secondary Education.

**Table 3:** Logistic regression predicting the types of volunteering during the COVID-19 pandemic in the UK (weighted; N = 31,890)

	Formal volunteering		Social action volunteering		Neighbourhood volunteering	
	OR	95% CI	OR	95% CI	OR	95% CI
**Model 1: demographic backgrounds**						
Age	1.02	1.00–1.05	1.00	0.98–1.01	**1.13**	**1.11–1.15**
Age-squared	**1.00**	**1.00–1.00**	1.00	1.00–1.00	**1.00**	**1.00–1.00**
Female (reference: male)	1.08	0.97–1.21	**1.31**	**1.22–1.41**	**1.20**	**1.11–1.29**
White ethnic (reference: ethnic minority)	**0.65**	**0.52–0.80**	0.92	0.78–1.08	1.11	0.94–1.31
Single and never married	1.08	0.88–1.32	1.00	0.87–1.14	**1.28**	**1.12–1.47**
Divorced or widowed	1.03	0.84–1.27	0.95	083–1.09	**1.25**	**1.09–1.44**
In a relationship/married but living apart (reference: in a relationship/married and cohabiting)	0.98	0.78–1.23	1.00	0.85–1.18	**1.68**	**1.43–1.98**
Living alone (reference: not living alone)	0.97	0.80–1.17	**0.84**	**0.75–0.95**	**0.54**	**0.48–0.61**
Number of children in the household	0.98	0.92–1.05	**0.95**	**0.91–0.99**	**1.16**	**1.10–1.21**
Living in city/town (reference: living in village/hamlet/isolated dwelling)	**0.80**	**0.72–0.90**	0.95	0.88–1.03	**0.88**	**0.81–0.95**
Constant	**0.15**	**0.08–0.32**	1.33	0.86–2.05	**0.05**	**0.03–0.09**
**Model 2: Model 1** + **socio-economic position**						
Full-time employment/self-employed	0.97	0.70–1.36	**1.39**	**1.10–1.76**	0.98	0.77–1.24
Part-time employment	1.16	0.83–1.64	**1.55**	**1.21–1.99**	1.06	0.83–1.36
Student/retired/homemakers/unable to work due to disability (reference: unemployed and seeking work)	1.20	0.86–1.67	**1.52**	**1.20–1.93**	0.92	0.72–1.17
Degree or above	**2.36**	**1.70–3.29**	**1.97**	**1.64–2.36**	1.12	0.92–1.36
A-levels	**1.72**	**1.22–2.44**	**1.46**	**1.20–1.77**	**1.33**	**1.08–1.63**
GCSE/post 16 vocational qualification (reference: no qualification)	**1.54**	**1.10–2.16**	1.17	0.98–1.42	**1.25**	**1.03–1.53**
Household income >£30,000 (reference: household income <£30,000)	1.07	0.93–1.22	**1.34**	**1.23–1.46**	1.01	0.93–1.10
Standard room/space households (reference: overcrowded households)	0.99	0.70–1.42	1.18	0.91–1.52	0.98	0.76–1.27
Keyworkers (reference: not keyworkers)	**1.45**	**1.25–1.68**	0.96	0.87–1.05	**1.34**	**1.23–1.47**
Constant	**0.06**	**0.03–0.13**	**0.31**	**0.17–0.54**	**0.06**	**0.03–0.10**
**Model 3: Model 2** + **Big five personalities**						
Extraversion	**1.29**	**1.22–1.36**	**1.22**	**1.18–1.26**	**1.15**	**1.11–1.19**
Neuroticism	0.95	0.90–1.01	**1.04**	**1.00–1.08**	**0.94**	**0.90–0.97**
Openness	**1.21**	**1.14–1.28**	**1.23**	**1.19–1.28**	**1.06**	**1.02–1.10**
Conscientiousness	1.00	0.94–1.05	1.03	0.99–1.07	**1.06**	**1.02–1.10**
Agreeableness	**1.10**	**1.04–1.16**	**1.14**	**1.10–1.18**	**1.09**	**1.05–1.13**
Constant	**0.05**	**0.02–0.11**	**0.26**	**0.15–0.47**	**0.05**	**0.03–0.10**
**Model 4: Model 2** + **psychosocial measures**						
Social support	**1.01**	**1.00–1.02**	**1.02**	**1.02–1.03**	**1.02**	**1.02–1.03**
Social network	**1.47**	**1.28–1.68**	**1.54**	**1.42–1.68**	**1.32**	**1.21–1.44**
Diagnosed mental health condition	**1.23**	**1.07–1.42**	**1.16**	**1.05–1.28**	1.03	0.94-1.14
Diagnosed physical health condition or disability	0.98	0.87–1.09	**1.08**	**1.00–1.17**	**0.73**	**0.67–0.79**
Constant	**0.03**	**0.01–0.08**	**0.12**	**0.07–0.22**	**0.03**	**0.01–0.05**

OR: odds ratio; CI: confidence interval; GCSE: General Certificate of Secondary Education. Bold values denote the statistical significance at the *p* < .05 level.

**Table 4:** Frequency of volunteering in April/May during lockdown versus volunteering across June/July

	I have not done any volunteering in June/July	Less than during April/May	About the same as during April/May	More than during April/May
Less than usual (April/May vs prior to the pandemic)	75.4%	6.3%	10.6%	7.7%
About the same (April/May vs prior to the pandemic)	86.3%	4.0%	7.5%	2.3%
More than usual (April/May vs prior to the pandemic)	52.6%	21.6%	18.8%	7.0%

## Data Availability

Anonymous data will be made available following the end of the pandemic.
