# Supplementary material for: Predictors of engaging in voluntary work during the COVID-19 pandemic: analyses of data from 31,890 adults in the UK
Source: Perspect Public Health. Author manuscript; Available in PMC 2022 Sep 23. (PMC9483687; doi:10.1177/1757913921994146)
Supplement: Supplementary Material [file EMS150147-supplement-Supplementary_Material.docx]

Supplementary Material

# Methods

To understand how types of volunteering varied across personal characteristics and backgrounds, we considered a rich set of demographic factors, socio-economic factors, personality traits, and psychosocial factors including respondents’ age, gender, ethnicity (white ethnic vs ethnic minority), partnership status (single and never married vs divorced or widowed vs in a relationship/married but living apart vs in a relationship/married and cohabiting), living arrangement (living alone vs not living alone), number of children in the household and living area (living in city/town vs living in village/hamlet/isolated dwelling). Socio-economic factors included employment status (full-time employment/self-employed vs part-time employment vs student/retired/homemakers/unable to work due to disability vs unemployed and seeking work), educational level (degree or above vs A-levels vs GCSE/post-16 vocational qualification vs no qualification), household income (income >£30,000 vs income <£30,000), housing space (standard room/space households vs overcrowded households) and whether respondents were keyworkers. Our model also considered the Big 5 personalities which are comprised of extraversion, neuroticism, openness, conscientiousness and agreeableness (derived from 15 items using factor analysis). Lastly, we included two psychosocial measures. Social support was measured using an adapted version of the six-item short form of Perceived Social Support Questionnaire (F-SozU K-6). Each item is rated on a 5-point scale from “not true at all” to “very true”, with higher scores indicating higher levels of perceived social support. Minor adaptations were made to the language in the scale to make it relevant to experiences during COVID-19 (see Supplementary Table 1 for a comparison of changes). Size of social network was measured by asking participants how many close friends they had, with responses dichotomised into <3 friends vs 3+ friends. We also asked participants if they had any diagnosed mental health conditions (including clinically-diagnosed depression, anxiety, or other mental health conditions), or any diagnosed physical condition or disability (including high blood pressure, diabetes, heart disease, lung disease, cancer, or other physical health conditions/disability).

**Supplementary Table 1: Comparison of items in the original and revised Perceived Social Support Questionnaire (F-SozU K-6).**

| Original | Adapted for COVID-19  In the past week, I feel… |
| --- | --- |
| I experience a lot of understanding and security from others | I have experienced a lot of understanding and support from others |
| I know a very close person whose help I can always count on | I have a very close person whose help I can always count on |
| If necessary, I can easily borrow something I might need from neighbours or friends | If necessary, I can easily borrow something I need from neighbours or friends |
| I know several people with whom I like to do things | I have people with whom I can spend time and do things together |
| When I am sick, I can without hesitation ask friends and family to take care of  important matters for me | If I get sick, I have friends and family who will take care of me |
| If I am down, I know to whom I can go without hesitation | If I am feeling down, I have people I can talk to without hesitation |

| **Supplementary Table 2 Logistic regression predicting the types of volunteering: keyworkers are excluded (weighted; N=24,564)** | | | | | | |
| --- | --- | --- | --- | --- | --- | --- |
|  | **Formal volunteering** | | **Social action volunteering** | | **Neighbourhood volunteering** | |
|  | **OR** | **95% CI** | **OR** | **95% CI** | **OR** | **95% CI** |
| *Model 1: Demographic backgrounds* | | | | |  |  |
| Age | 1.03 | 1.00 - 1.06 | 1.00 | 0.98 - 1.02 | **1.12** | **1.10 - 1.14** |
| Age-squared | **1.00** | **1.00 - 1.00** | 1.00 | 1.00 - 1.00 | **1.00** | **1.00 - 1.00** |
| Female | **1.15** | **1.01 - 1.30** | **1.34** | **1.24 - 1.46** | **1.15** | **1.06 - 1.25** |
| (ref: male) |  |  |  |  |  |  |
| White ethnic | **0.72** | **0.56 - 0.92** | 0.98 | 0.81 - 1.18 | 1.09 | 0.90 - 1.32 |
| (ref: ethnic minority) |  |  |  |  |  |  |
| Single and never married | 0.88 | 0.69 - 1.11 | 0.92 | 0.79 - 1.07 | **1.27** | **1.09 - 1.48** |
| Divorced or widowed | 0.85 | 0.68 - 1.06 | 0.90 | 0.77 - 1.04 | **1.19** | **1.02 - 1.39** |
| In a relationship/ married but living apart | 0.80 | 0.61 - 1.06 | 0.93 | 0.77 - 1.13 | **1.60** | **1.32 - 1.94** |
| (ref: in a relationship/married and cohabiting) |  |  |  |  |  |  |
| Living alone | 1.15 | 0.92 - 1.43 | 0.88 | 0.76 - 1.01 | **0.55** | **0.48 - 0.64** |
| (ref: not living alone) |  |  |  |  |  |  |
| Number of children in the household | 0.95 | 0.88 - 1.03 | **0.94** | **0.89 - 0.99** | **1.14** | **1.08 - 1.20** |
| Living in city/town | **0.79** | **0.69 - 0.91** | 0.96 | 0.87 - 1.05 | **0.89** | **0.81 - 0.98** |
| (ref: living in village/hamlet/isolated dwelling) |  |  |  |  |  |  |
| Constant | **0.12** | **0.06 - 0.27** | 1.18 | 0.73 - 1.92 | **0.07** | **0.04 - 0.11** |
| *Model 2: Model 1 + Socio-economic position* | | |  |  |  |  |
| Full-time employment/self employed | 1.04 | 0.75 - 1.44 | **1.38** | **1.09 - 1.75** | 0.98 | 0.77 - 1.24 |
| Part-time employment | 1.25 | 0.88 - 1.77 | **1.56** | **1.21 - 2.03** | 1.09 | 0.84 - 1.42 |
| Student/ retired/ homemakers/ unable to work due to disability | 1.16 | 0.84 - 1.62 | **1.47** | **1.15 - 1.87** | 0.93 | 0.73 - 1.19 |
| (ref: unemployed & seeking work) |  |  |  |  |  |  |
| Degree of above | **2.30** | **1.61 - 3.29** | **1.93** | **1.59 - 2.34** | 1.06 | 0.86 - 1.29 |
| A-levels | **1.66** | **1.14 - 2.43** | **1.41** | **1.15 - 1.73** | **1.26** | **1.02 - 1.57** |
| GCSE/post-16 vocational qualification | 1.40 | 0.97 - 2.01 | 1.12 | 0.92 - 1.36 | 1.12 | 0.91 - 1.37 |
| (ref: no qualification) |  |  |  |  |  |  |
| Household income >£30,000 | 1.08 | 0.93 - 1.25 | **1.36** | **1.23 - 1.49** | 1.04 | 0.94 - 1.14 |
| (ref: household income <£30,000) |  |  |  |  |  |  |
| Standard room/space households | 1.18 | 0.76 - 1.82 | 1.30 | 0.98 - 1.71 | 1.12 | 0.84 - 1.49 |
| (ref: overcrowded households) |  |  |  |  |  |  |
| Constant | **0.04** | **0.02 - 0.10** | **0.26** | **0.14 - 0.48** | **0.06** | **0.03 - 0.11** |
| *Model 3: Model 2 + Big 5 personalities* | | |  |  |  |  |
| Extraversion | **1.29** | **1.21 - 1.37** | **1.21** | **1.16 - 1.26** | **1.16** | **1.12 - 1.21** |
| Neuroticism | 0.96 | 0.91 - 1.03 | 1.04 | 1.00 - 1.09 | **0.92** | **0.89 - 0.97** |
| Openness | **1.23** | **1.15 - 1.31** | **1.25** | **1.20 - 1.30** | **1.05** | **1.01 - 1.10** |
| Conscientiousness | 1.00 | 0.94 - 1.06 | **1.04** | **1.00 - 1.09** | **1.08** | **1.04 - 1.12** |
| Agreeableness | **1.11** | **1.04 - 1.18** | **1.16** | **1.11 - 1.21** | **1.08** | **1.04 - 1.13** |
| Constant | **0.03** | **0.01 - 0.09** | **0.22** | **0.12 - 0.41** | **0.05** | **0.03 - 0.10** |
| *Model 4: Model 2 + Psychosocial measures* | |  |  |  |  |  |
| Social support | 1.01 | 1.00 - 1.02 | **1.02** | **1.02 - 1.03** | **1.02** | **1.02 - 1.03** |
| Social network | **1.54** | **1.33 - 1.79** | **1.52** | **1.39 - 1.67** | **1.34** | **1.21 - 1.48** |
| Diagnosed mental health condition | **1.33** | **1.13 - 1.57** | **1.17** | **1.05 - 1.31** | 1.02 | 0.91 - 1.14 |
| Diagnosed physical health condition or disability | 0.89 | 0.79 - 1.01 | **1.10** | **1.01 - 1.19** | **0.73** | **0.67 - 0.80** |
| Constant | **0.02** | **0.01 - 0.06** | **0.10** | **0.05 - 0.20** | **0.02** | **0.01 - 0.05** |
| Notes: Bold values denote statistical significance at the p < 0.05 level. | | | | | | |

| **Supplementary Table 3 Multinomial logistic regression predicting the amount of volunteering in the past month during the COVID-19 pandemic compared to usual amount (prior to the pandemic) (weighted; N=31,890)** | | | | |
| --- | --- | --- | --- | --- |
|  | **Less than usual vs about the same as usual** | | **More than usual vs about the same as usual** | |
|  | **RRR** | **95% CI** | **RRR** | **95% CI** |
| *Model 1: Demographic backgrounds* |  |  |  |  |
| Age | **0.95** | **0.93 - 0.97** | **1.08** | **1.05 - 1.11** |
| Age-squared | **1.00** | **1.00 - 1.00** | **1.00** | **1.00 - 1.00** |
| Female | **1.51** | **1.38 - 1.66** | **1.26** | **1.13 - 1.41** |
| (ref: male) |  |  |  |  |
| White ethnic | **0.60** | **0.49 - 0.73** | 0.83 | 0.66 - 1.03 |
| (ref: ethnic minority) |  |  |  |  |
| Single and never married | 1.15 | 0.99 - 1.35 | 1.04 | 0.85 - 1.27 |
| Divorced or widowed | **1.24** | **1.06 - 1.45** | 1.09 | 0.88 - 1.35 |
| In a relationship/ married but living apart | **1.32** | **1.09 - 1.60** | 0.97 | 0.76 - 1.24 |
| (ref: in a relationship/married and cohabiting) |  |  |  |  |
| Living alone | 0.97 | 0.84 - 1.12 | 0.91 | 0.75 - 1.10 |
| (ref: not living alone) |  |  |  |  |
| Number of children in the household | 1.03 | 0.97 - 1.09 | 0.96 | 0.90 - 1.03 |
| Living in city/town | 0.96 | 0.87 - 1.05 | **0.81** | **0.72 - 0.92** |
| (ref: living in village/hamlet/isolated dwelling) |  |  |  |  |
| Constant | 0.85 | 0.49 - 1.46 | **0.04** | **0.02 - 0.09** |
| *Model 2: Model 1 + Socio-economic position* |  |  |  |  |
| Full-time employment/self employed | **0.59** | **0.44 - 0.79** | 1.00 | 0.71 - 1.41 |
| Part-time employment | 0.84 | 0.62 - 1.14 | 1.20 | 0.83 - 1.72 |
| Student/ retired/ homemakers/ unable to work due to disability | 1.02 | 0.76 - 1.36 | 1.07 | 0.75 - 1.53 |
| (ref: unemployed & seeking work) |  |  |  |  |
| Degree of above | 1.21 | 0.99 - 1.49 | **1.97** | **1.41 - 2.75** |
| A-levels | 1.01 | 0.81 - 1.26 | **1.49** | **1.05 - 2.11** |
| GCSE/post-16 vocational qualification | 0.94 | 0.76 - 1.16 | 1.31 | 0.93 - 1.84 |
| (ref: no qualification) |  |  |  |  |
| Household income >£30,000 | 0.92 | 0.83 - 1.03 | 1.11 | 0.98 - 1.26 |
| (ref: household income <£30,000) |  |  |  |  |
| Standard room/space households | 0.76 | 0.56 - 1.03 | 1.41 | 0.95 - 2.10 |
| (ref: overcrowded households) |  |  |  |  |
| Keyworkers | 0.96 | 0.85 - 1.09 | 0.91 | 0.80 - 1.04 |
| (ref: not keyworkers) |  |  |  |  |
| Constant | 0.84 | 0.43 - 1.66 | **0.01** | **0.01 - 0.04** |
| *Model 3: Model 2 + Big 5 personalities* |  |  |  |  |
| Extraversion | **1.15** | **1.10 - 1.20** | **1.24** | **1.18 - 1.31** |
| Neuroticism | **1.07** | **1.02 - 1.12** | 1.03 | 0.97 - 1.09 |
| Openness | **1.09** | **1.04 - 1.14** | **1.19** | **1.12 - 1.26** |
| Conscientiousness | 1.00 | 0.96 - 1.05 | 1.04 | 0.99 - 1.10 |
| Agreeableness | **1.05** | **1.00 - 1.09** | **1.10** | **1.05 - 1.17** |
| Constant | 0.74 | 0.37 - 1.45 | **0.01** | **0.00 - 0.03** |
| *Model 4: Model 2 + Psychosocial measures* |  |  |  |  |
| Social support | **0.99** | **0.98 - 0.99** | **1.01** | **1.01 - 1.02** |
| Social network | **1.50** | **1.35 - 1.67** | **1.51** | **1.34 - 1.71** |
| Diagnosed mental health condition | **1.17** | **1.04 - 1.31** | **1.23** | **1.07 - 1.41** |
| Diagnosed physical health condition or disability | **1.17** | **1.07 - 1.28** | 0.91 | 0.82 - 1.02 |
| Constant | 0.86 | 0.42 - 1.77 | **0.01** | **0.00 - 0.02** |
| Notes: Bold values denote statistical significance at the p < 0.05 level. | | | | |
